# Spatial and Temporal Dynamics of Contact Zones Between Chromosomal Races of House Mice, *Mus musculus domesticus,* on Madeira Island

**DOI:** 10.3390/genes11070748

**Published:** 2020-07-06

**Authors:** Joaquim T. Tapisso, Sofia I. Gabriel, Ana Mota Cerveira, Janice Britton-Davidian, Guila Ganem, Jeremy B. Searle, Maria da Graça Ramalhinho, Maria da Luz Mathias

**Affiliations:** 1 CESAM – Centro de Estudos do Ambiente e do Mar, Faculdade de Ciências, Universidade de Lisboa, 1749-016 Lisboa, Portugal; jstapisso@fc.ul.pt (J.T.T.); sigabriel@fc.ul.pt (S.I.G.); ana.cerveira@gmail.com (A.M.C.); gramalhinho@fc.ul.pt (M.d.G.R.); 2 Departamento de Biologia, Universidade de Aveiro, Campus Universitário de Santiago, 3810-193 Aveiro, Portugal; 3 ISEM – Institut des Sciences de l’Evolution de Montpellier, Université de Montpellier, CNRS, EPHE, IRD, 34095 Montpellier, France; mlmathias@fc.ul.p (J.B.-D.); Guila.Ganem@umontpellier.fr (G.G.); 4 Department of Ecology and Evolutionary Biology, Corson Hall, Cornell University, Ithaca, NY 14853, USA; jeremy.searle@cornell.edu

**Keywords:** Robertsonian fusions, chromosomal evolution, distribution, clinal analysis

## Abstract

Analysis of contact zones between parapatric chromosomal races can help our understanding of chromosomal divergence and its influence on the speciation process. Monitoring the position and any movement of contact zones can allow particular insights. This study investigates the present (2012–2014) and past (1998–2002) distribution of two parapatric house mouse chromosomal races—PEDC (Estreito da Calheta) and PADC (Achadas da Cruz)—on Madeira Island, aiming to identify changes in the location and width of their contact. We also extended the 1998–2002 sampling area into the range of another chromosomal race—PLDB (Lugar de Baixo). Clinal analysis indicates no major geographic alterations in the distribution and chromosomal characteristics of the PEDC and PADC races but exhibited a significant shift in position of the Rb (7.15) fusion, resulting in the narrowing of the contact zone over a 10+ year period. We discuss how this long-lasting contact zone highlights the role of landscape on mouse movements, in turn influencing the chromosomal characteristics of populations. The expansion of the sampling area revealed new chromosomal features in the north and a new contact zone in the southern range involving the PEDC and PLDB races. We discuss how different interacting mechanisms (landscape resistance, behaviour, chromosomal incompatibilities, meiotic drive) may help to explain the pattern of chromosomal variation at these contacts between chromosomal races.

## 1. Introduction

Since the first study by Gropp et al. [1], numerous chromosomal races of the western house mouse (*Mus musculus domesticus*), resulting from centric or Robertsonian (Rb) fusions of pairs of acrocentric chromosomes, have been identified in Europe and North Africa [2,3]. Robertsonian races are defined by presence of metacentric chromosomes and chromosome numbers that can be reduced as low as 2n = 22, in contrast to the all-acrocentric 2n = 40 standard race [2]. Further chromosomal variability arises from the occurrence of whole-arm reciprocal translocations (WARTs) and through hybridisation [4,5]. Areas of contact between different chromosomal races have been extensively studied in the house mouse providing insights into the role of chromosomal variation on population divergence and speciation [3,5,6,7,8,9]. Specifically, it has been argued that chromosomal rearrangements may promote hybrid unfitness leading to reduced interracial gene flow close to the mutation breakpoint, as well as recombination suppression in the same genomic region. This can result in genetic divergence, ultimately leading to ’parapatric speciation’, i.e., the situation where, in a geographical context, two neighbouring forms/races become separate species whilst in contact and hybridising [8,10,11,12]. Additionally, behavioural factors, such as divergence of mate recognition systems, environmental factors, such as barriers to dispersal, and stochastic factors, resulting from source/sink demographic population systems, may also contribute to decreased gene flow, thus enhancing reproductive isolation between the hybridising races [5,13,14,15,16,17,18,19,20,21,22].

The majority of chromosomal races in the house mouse are grouped into metacentric ‘systems’ occupying defined geographical areas. These enable contact and hybridisation between chromosomal races with different sets of metacentric chromosomes, but also between those metacentric races and the standard all-acrocentric race that surrounds the ‘system’ [2]. At present, about 20 hybrid zones have been described, which are discrete contacts between chromosomal races as well as some polymorphic areas referred to as unconfirmed hybrid zones, e.g. [3].

**Figure 1:**
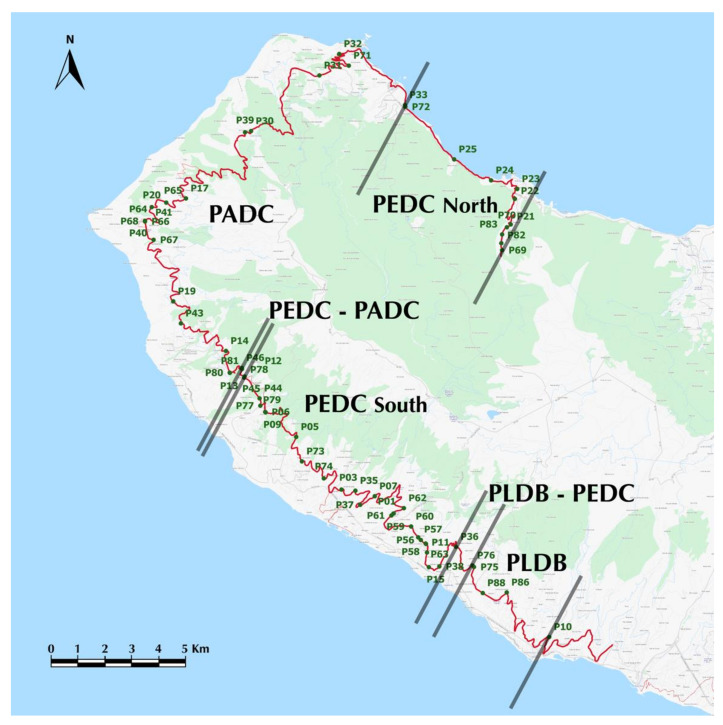
Location of sampling sites (dark green dots) orthogonally projected on the roads ER222 and ER101 (red line) of Madeira. See Table S1 for details on sampling sites. Black lines represent the limits of different chromosomal zones, PLDB represents the Lugar de Baixo race, PLDB-PEDC the contact zone between the Lugar de Baixo and Estreito da Calheta races, PEDC the Estreito da Calheta race, PEDC-PADC the contact zone between the Estreito da Calheta and Achadas da Cruz races and PADC the Achadas da Cruz race.

The Madeira ‘system’, characterising the western house mouse populations on the island of Madeira, involves an extraordinary chromosomal variability and includes six metacentric races with diploid numbers ranging from 22 to 38 [4,23]. So far, a single contact zone between two neighbouring parapatric races – the PEDC race (Estreito da Calheta) and the PADC race (Achadas da Cruz) [17] – has been found in a previous survey (1998–2002). The two races share seven Rb fusions (2n = 24) and differ by the occurrence of Rb (6.7) in the PEDC race and Rb (7.15) in PADC [23]. A notable characteristic of this zone is the staggered clines of the diagnostic fusions due to the presence of chromosome 7 in an acrocentric state, e.g. [5]. Human activities influencing the wider environment were proposed to be the main factors regulating the structure and location of this zone [17]. These two races occur in the westernmost part of Madeira, the PEDC race in the southwest plus in an apparently isolated area in the north coast, and the PADC race in a more north-westward area, located between the main distributional area of PEDC and its isolated population in the north (Figure 1). 

In the present study, based on a recent survey (2012-2014), we reanalyse the distribution of the PADC and PEDC races and the extent and structure of the previously identified contact zone on the south coast of Madeira. We also extend the sampling area northwards to investigate the northern contact between the PEDC and PADC races. Furthermore, we include a third race (Lugar de Baixo, PLDB) in the analysis, occurring eastward of the southern range of PEDC and separated from this race by a 2 km-wide area of natural vegetation [17], that may represent a natural barrier for active dispersal of house mice. The PLDB race also shares a total of seven fusions (2n = 24) with the PEDC race, again only differing in one, Rb (15.18) vs. (5.18), respectively [23]. 

Most of the studies carried out on the structure of contact zones involving Rb races of *M. m. domesticus* have described the contact of two freely interacting populations, such as PADC and PEDC. Few studies have considered the influence of physical barriers on hybridising forms (e.g. [24] and references therein). Here we aimed to investigate: a) the structure of the contact zone between the PEDC and PADC races, by comparing the results from two surveys conducted over ten years apart, b) the chromosomal features of the northern populations of mice, that could not be analysed in the 1998–2002 survey because of unsuccessful trapping, and c) the effect of both environmental and physical barriers on mouse dispersal and the influence they may have on the chromosomal characteristics of the PEDC-PADC and PEDC-PLDB contact zones. Previously it was hypothesised that PEDC and PADC occupied habitats differed in quality, providing a basis for source-sink dynamics to emerge [17]. Spatial differentiation in habitat quality is the major factor stabilising source-sink systems, although other factors may also affect movements of individuals among populations, e.g. [25,26,27]. By attaining the above goals we expect to answer the following questions: i) has the contact zone between the PEDC and PADC races moved or changed in width?, ii) do populations of the PADC race and the northern isolated PEDC population interact?, iii) can source-sink dynamics explain the distribution of PEDC and PLDB and the movement of mice between these populations? iv) is there a natural barrier that could block active dispersal of house mice in this system (therefore influencing chromosomal characteristics)?

By answering these questions, we hope to better understand to what extent the chromosomal races studied are physically isolated and the potential impact of interaction or hybridisation between the races on further reproductive isolation and divergence.

## 2. Material and Methods

### 2.1. Study Area and Sampling of Mice

A total of five field trips to Madeira Island, each lasting approximately three weeks, were carried out between 2012 and 2014. Sampling of house mice took place across the geographical range of three metacentric races, Lugar de Baixo (hereafter PLDB), Estreito da Calheta (hereafter PEDC) and Achadas da Cruz (hereafter PADC) [17,23]. The races were named following Ramalhinho et al. [28] and Piálek et al. [2] and are all characterised by eight Rb fusions differing from each other by single fusions [17,23]. PLDB differs from PEDC by the presence of Rb (15.18) and absence of Rb (5.18) fusions, while PEDC differs from PADC by the presence of Rb (6.7) and absence of Rb (7.15) fusions. The combination of chromosomal fusions displayed by each race is as follows:

PLDB: Rb (2.4) (3.14) (6.7) (8.11) (9.12) (10.16) (13.17) (15.18)

PEDC: Rb (2.4) (3.14) (5.18) (6.7) (8.11) (9.12) (10.16) (13.17) 

PADC: Rb (2.4) (3.14) (5.18) (7.15) (8.11) (9.12) (10.16) (13.17) 

Based on the habitat preferences of the house mouse in Madeira [17], sampling sites were mainly located in human dwellings and human-modified habitats (farms, cultivated and fallow fields). Altogether, we sampled 65 sites along a south-northwest-east transect c. 83 km long, between the villages of Ponta do Sol and Chão da Ribeira (Figure 1; Table S1). The sites were numbered sequentially from site 1 at the southern end, to site 65 at the north-eastern end of the transect. Sites 6 to 50 correspond approximately to the sites previously surveyed by Nunes et al. [17] over the period 1998–2002. The distance between sampling sites ranged between c. 300 m and 8 km, according to terrain (Figure 1). All animals were captured in Sherman live-traps baited with sardine paste. Traps were set for one to three nights depending on trapping success. Mice were transported to animal facilities in Funchal (“Estação de Biologia Marinha”) for karyotyping and data gathering. All applicable international, national, and/or institutional guidelines for the care and use of animals were followed. Capturing and testing were conducted by researchers certified by Ministério da Agricultura, do Mar do Ambiente e do Ordenamento do Território (01/2014/CAPT) for Portugal.

### 2.2. Chromosome Analysis

Karyotypes were prepared from bone marrow cells, following standard protocols used in earlier studies [4], namely the ‘air-drying’ procedure [29] and G-banding of chromosomes [30]. A minimum of three metaphases per individual were analysed, making sure that all homologous pairs of chromosomes were identified in each. Metaphases were observed under an Olympus microscope and karyotyped using Leica Chantal software. Chromosomes were identified following Cowell [31] at the Faculty of Sciences in Lisbon and at the Institute of Evolutionary Sciences, Montpellier. Chromosomal analyses were performed using an Olympus BX41 microscope with an attached Leica DC 250 camera equipped with a Leica CW4000 Karyo image analysis system. 

The distribution of metacentric races was recorded following the methodology described in Nunes et al. [17]. Accordingly, the sampling sites were distributed along the main road running around the western region of Madeira Island (ER222 and ER101, Figure 1), because most houses were there and it likely acts as the dispersal route for mice. Excluding two sampling points, located 1250 m and 1750 m from the road, all other sites were located c. 2m to 865 m away from the road. In order to fully compare the results from both surveys (1998–2002 vs. 2012-2014), the exact same methodology was employed. As such, the positions of sampling sites off the main road were orthogonally projected onto it, see [17]. Sites located less than 200 m apart after projection were pooled and treated as a single site in all analyses. For each site, we: i) determined the diploid number of all animals, ii) identified all fusions, and iii) determined the percentage of acrocentric and metacentric chromosomes for diagnostic chromosomes and fusions. 

### 2.3. Temporal Analysis

For the temporal analysis, we compared our new dataset with the one obtained between 1998 and 2002 (Table S2). Previously sampled sites were re-projected orthogonally along the main road to avoid errors when comparing distances among sites between the two periods. The 1998–2002 dataset includes the results reported by Nunes et al. [17] and unpublished data (Table S2). Temporal comparisons included: i) the chromosomal characteristics of sites and groups of sites for the whole transect, ii) the percentage of acrocentrics and metacentrics in sites and groups of sites also for the whole extension of the transect, and iii) the clinal pattern for diagnostic fusions of PEDC (Rb 6.7) and PADC (Rb 7.15) along the previously sampled transect analysed by Nunes et al. [17], i.e., sites 6 to 50 (km 10.1 to 58.17). The software package C-Fit8 was used to compute eight regression cline models (Table S3). Model selection was then performed based on the Akaike’s information criterion [32] (Table S3). Logistic or scaled logistic functions were selected to best fit the metacentric clines:

f(x) = (e^s(x−c)^/1 + e^s(x−c)^) × h, (1)

where *x* is the geographic distance, *s* and *c* are the maximum slope and the centre of the cline, respectively, and *h* is the height. The width (expressed as an inverse of the slope) and cline centres were compared between the pair of fusions Rb (6.7) and Rb (7.15) for both time periods. To check for differences between clines, we assumed that twice the difference in log_e_ likelihood values between the constrained and unconstrained model (on cline centre for coincidence and on cline width for concordance) followed a χ^2^ distribution with a *d.f.* equal to the difference of the parameters estimated, see [33].

## 3. Results 

### 3.1. Current Chromosomal Variation in Western Madeira 

After projecting all sampling sites on the road (Figure 1), 29 sites were pooled (in combinations of 2 to 4 sites). Pooling of sites restricted chromosomal analyses to a total of 48 sites. A total of 449 mice were trapped along the transect, 363 of which were successfully karyotyped. The number of karyotyped animals per site ranged from 1 to 31. Each site was allocated to a zone/race on the basis of frequency of the race-specific Rb fusions, i.e., Rb (15.18) vs. (5.18) and Rb (6.7) vs. (7.15) (Figure 1).

Fusion Rb (15.18) was identified along the first six sites of the transect (site 1 to 6 at km 0 to 10.1). From sites 1 to 4 (km 0 to 8.4) all mice carried this fusion in a homozygous state, and in sites 5 and 6 (km 8.48 and 10.1) in a heterozygous state as a result of hybridisation between PEDC and PLDB.

**Figure 2:**
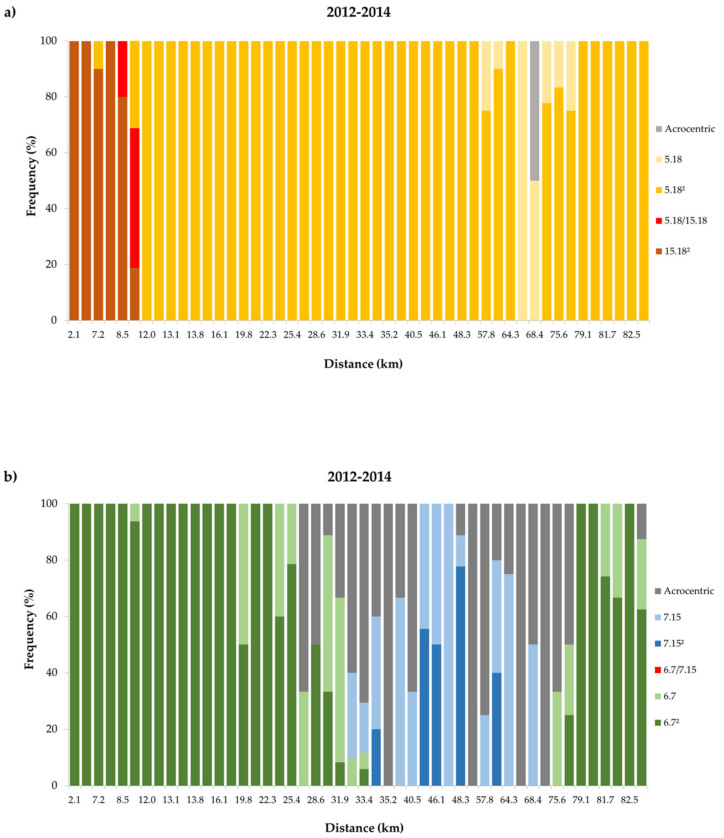
Frequency of diagnostic Rb fusions for the period 2012-2014. a) the upper panel shows frequencies of fusions Rb (15.18) and Rb (5.18) in homozygous [(15.18)^2^; (5.18)^2^] and heterozygous [(5.18)] state, frequency of hybrid mice carrying both fusions [(5.18)/(15.18)] and frequencies of mice carrying none of the fusions (Acrocentric). b) the lower panel shows frequencies of fusions Rb (6.7) and Rb (7.15) in homozygous [(6.7)^2^; (7.15)^2^] and heterozygous [(6.7); (7.15)] states, frequency of hybrid mice carrying both fusions [(6.7)/(7.15)] and frequencies of mice carrying none of the fusions (Acrocentric).

**Figure 3:**
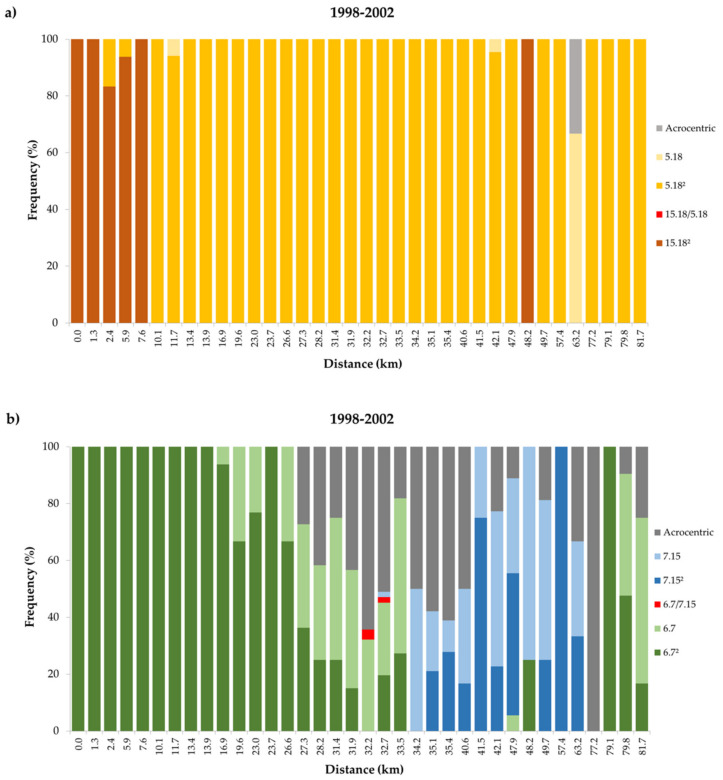
Frequency of diagnostic chromosomal fusions for the period 1998–2002. a) the upper panel shows frequencies of fusions Rb (15.18) and Rb (5.18) in homozygous [(15.18)^2^; (5.18)^2^] and heterozygous [(5.18)] states, frequency of hybrid mice carrying both fusions [(5.18)/(15.18)] and frequencies of mice carrying none of the fusions (Acrocentric). b) the lower panel shows frequencies of fusions Rb (6.7) and Rb (7.15) in homozygous [(6.7)^2^; (7.15)^2^] and heterozygous [(6.7); (7.15)] states, frequency of hybrid mice carrying both fusions [(6.7)/(7.15)] and frequencies of mice carrying none of the fusions (Acrocentric).

Fusion Rb (5.18), in a homozygous state, first occurred in a mouse at site 3 (km 7.16) and again at sites 5 and 6 (km 8.48 and 10.1), both in a homozygous state (5 out of 21 mice) and a heterozygous state (10 out of 21 mice). These heterozygous Rb (5.18) mice were the same that also carried the fusion Rb (15.18) in a heterozygous state mentioned above, again indicating the occurrence of hybridisation. From site 7 to site 48 (km 12 to 49.54) all mice carried the fusion Rb (5.18) in a homozygous state (211 mice). Exceptionally, at site 32 (km 32.95) chromosomes 5 and 18 were acrocentric in one of the sampled mice. From sites 49 to 57 (km 57.83 to 77.36) the fusion occurred in both homozygous (49 out of 58 mice) and heterozygous states (9 out of 58 mice). Along the last sites on the transect (site 58 to 65 at km 79.12 to 82.78) fusion Rb (5.18) was again homozygous (i.e., fixed) in all mice sampled (58 individuals) (Figure 2; Figure 3).

Fusion Rb (6.7) was homozygous in most animals collected from sites 1 to 18 (km 0 to 16.63) (96 out of 97 mice), with the exception of one heterozygous mouse for Rb (6.7) at site 6 (km 10.1). From site 19 (km 19.85) northwards the frequency of occurrence of this fusion gradually decreased, being absent between sites 35 to 55 (km 33.47 to 72.72) but occurring again between sites 56 and 65 (km 75.59 to 82.78) (51 out of 67 mice). In this section of the transect the fusion occurred both in a homozygous or heterozygous state. 

Fusion Rb (7.15) occurred in mice sampled from sites 33 to 53 (km 33 to 68.39), usually in a heterozygous state. Mice with acrocentric chromosomes 6, 7, and 15 co-occurred with mice carrying both Rb (6.7) and Rb (7.15) fusions from site 25 (km 28.6) until the end of the transect (km 82.78).

Based on the above description, it was possible to classify different segments of the transect according to the relative frequency of the four diagnostic Rb fusions (Figure 1 and Figure 2). Animals assigned to the PLDB race occurred in the first segment on the south coast of Madeira (sites 1 to 6 at km 0 to 10.1). The identification of PLDB-PEDC hybrids in the last two sites of this segment (sites 5 to 6 at km 8.48 to 10.1) confirmed a hybrid zone between the two races. The next segment is characterised by animals carrying the PEDC diagnostic fusions in a homozygous state (sites 7 to 18 at km 12 to 16.63), followed by a longer segment where animals carried the fusion Rb (6.7) in a heterozygous state (sites 19 to 34 at km 19.85 to 33.42). This segment is followed by a very small area, where PEDC and PADC overlap, although no hybrids between them were identified (sites 33 to 34 at km 33 to 33.42). The next segment occupies the entire north-west corner of Madeira Island (sites 35 to 53 at km 33.47 to 68.39), where individuals were either assigned to PADC, due to the presence of Rb (7.15), or carried the acrocentrics 6, 7, and 15. The last segment of the transect (sites 56 to 65 at km 75.59 to 82.78) was characterised by mice with Rb (6.7), either in a homozygous or heterozygous state. A few animals along this last section of the transect had acrocentrics 6, 7, and 15 (16 mice). 

### 3.2. Temporal Variation in the Distribution of Madeira Rb Races

The distribution of races PLDB, PEDC and PADC shows no significant differences between the two periods analysed (1998–2002 versus 2012-2014). Animals assigned to the race PLDB were found until km 10.1 (Figure 2a and Figure 3a). In this section of the transect, the main difference between temporal periods is the detection of hybrids between races PLDB and PEDC in the 2012-2014 sampling (Figure 2a). 

The next segment of the transect is characterised by mice assigned to race PEDC, with fusion Rb (6.7) in a homozygous state (Figure 2b and Figure 3b), in the period 1998–2002 between km 11.7 and 13.9 and in the period 2012-2014 between km 12 and 16.6.

The following segment, starting at km 16.9 in 1998–2002 and at km 19.8 in 2012-2014, was characterised by the presence of homozygous and heterozygous mice for Rb (6.7), and mice carrying acrocentric chromosomes 6 and 7. This segment ended at km 31.9 in both periods analysed. This was followed by a very short segment defined by the presence of mice carrying fusions Rb (6.7) or (7.15) either in homozygous or heterozygous states and mice acrocentric for the chromosomes involved in both fusions. 

Despite the substantial sampling effort in this segment, hybrids between races PEDC and PADC were exclusively found in the period 1998–2002. This segment was located between km 32.2 and 33.5 for the period 1998–2002 and between km 33.0 and 33.4 for the period 2012-2014. 

Adjacent to this short region, a longer segment, ranging from km 34.2 to km 63.2 in 1998–2002 and from km 34.2 to km 68.4 in 2012-2014, is characterised by the presence of mice homozygous or heterozygous for the fusion Rb (7.15) as well as mice acrocentric for chromosomes 7 and 15. 

The last segment of the transect was characterised by the presence of mice either homozygous or heterozygous for Rb (6.7), and mice carrying acrocentric chromosomes 6 and 7. For the period 1998–2002 this segment was located between km 77.2 and km 81.7, while for the period 2012-2014 it was located between km 72.7 and km 82.8. 

Following the above results we assessed the present extent and location of the different chromosomal zones (Figure 1): zone **PLDB**; zone **PLDB–PEDC,** a contact zone between the PLDB and PEDC races; zone **PEDC South**, including mice either homozygous or heterozygous for Rb (6.7) and mice carrying acrocentric 6 and 7; zone **PEDC–PADC,** a contact zone between the PEDC and PADC races; zone **PADC,** with mice either homozygous or heterozygous for Rb (7.15) plus mice acrocentric for 7 and 15; and zone **PEDC North,** with identical characteristics to zone PEDC South. 

**Figure 4:**
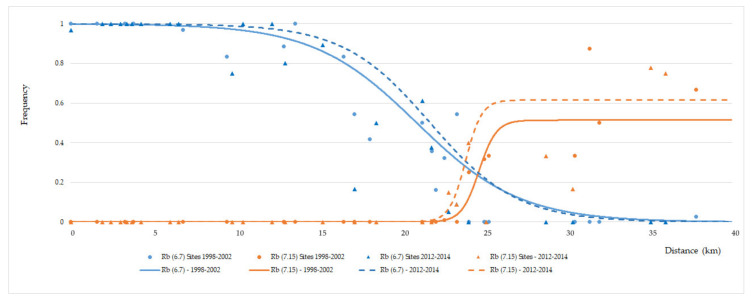
Comparison of clinal patterns for fusions Rb (6.7) (blue) and Rb (7.15) (orange) between the periods 1998–2002 (circles and full line) and 2012-2014 (triangles and dashed line).

Cline model estimates are summarised in Table 1. Tests of concordance and coincidence of the fusion Rb (6.7) revealed similarities between the two periods analysed (Figure 4). The results of maximum likelihood ratio tests revealed no significant differences in the cline width between years 1998–2002 and 2012-2014 (12.48 vs. 10.61, respectively; maximum likelihood difference of 1.43, *p* = 0.232), nor between cline centres (19.07 vs. 18.26; maximum likelihood difference of 1.70, *p* = 0.192). Cline analysis of the Rb (7.15) fusion also showed a similarity between cline width in the two periods analysed (2.09 vs. 1.96; maximum likelihood difference of 0.03, *p* = 0.862) but a significant difference between cline centres (24.71 vs. 23.89; maximum likelihood difference of 9.04, *p* = 0.003) was observed.

## 4. Discussion 

### 4.1. Current Distribution of Estreito da Calheta (PEDC) and Achadas da Cruz (PADC) Races

The possible involvement of chromosomal rearrangements in species formation has engendered much interest. Several models of speciation have been proposed, emphasising the role of hybrid zones between chromosomal races, e.g. [34,35]. The western house mouse, due to the accumulation and fixation of Robertsonian fusions, has been considered an excellent model to study how chromosomal rearrangements may be implicated in population divergence, race formation, and speciation, including the analysis of chromosomal hybrid zones [9]. Here, we compared the structure, position and width of the hybrid zone between two chromosomal races (PEDC and PADC) on Madeira Island at two time periods 10 years apart, and expanded the sampling efforts at both ends of the previously analysed transect.

Our results confirm the previous findings by Nunes et al. [17] of four chromosomal zones occurring along the south-western and northward distribution of the PEDC-PADC contact. As described in the previous study, the frequency of diagnostic fusions Rb (6.7) and Rb (7.15) varied along the transect. One of the main differences between the two time periods was the broadening of the PADC chromosomal zone, i.e., a wider geographic detection of mice assigned to this race, reflecting the increase of the sampling area during the 2012-2014 survey in the north-western part of the island. The increase of the transect along the north-western coast towards the northern side of the island also allowed another contact zone – PEDC North – to be identified between PADC and the previously described (and very geographically circumscribed) PEDC isolate on the north coast, characterised by the presence of mice mostly homozygous or heterozygous for Rb (6.7), with a few occasional mice exclusively acrocentric for these chromosomes.

Our 2012-2014 survey, particularly along the northern side of Madeira also revealed the occurrence of fusion Rb (1.15) whose distribution had not previously been identified on the island. This fusion, always found in a heterozygous state, appears to be more frequent in the PEDC North zone, having been detected in a total of 12 mice from 6 sampling sites. However, it was also very occasionally found outside this range, namely in the PEDC South zone (in two mice in a single location) and in the northernmost end of the PADC zone in a single mouse, heterozygous for Rb (7.15). In nature, chromosome 1 has been involved in several fusions, however Rb (1.15) is not a common one [2,36]. In Madeira, we suggest that Rb (1.15) may result from a recent mutation event, considering the fusion’s somewhat restricted geographic distribution and its exclusive heterozygous state. This fusion seems to have emerged multiple times in the house mouse in situations where chromosomes 1 and 15 are available to fuse but, so far, it has never been found in a fixed state. A similar situation has been suggested by Sans-Fuentes et al. [37] for the appearance of a rare fusion, Rb (7.17) in the polymorphic Barcelona ‘system’, and by Adolph & Klein [38] for several rare Rb fusions in mice from southern Germany. 

Our results suggest that some temporal change has occurred in the spatial distribution of the diagnostic fusions in the PEDC-PADC parapatric area, resulting in the narrowing of the contact zone between them. Comparisons involving Rb (6.7) indicate that the general clinal pattern for this fusion did not change significantly between 1998–2002 and 2012–2014, i.e., no significant variation was observed in either the cline centre or width. However, for Rb (7.15), a statistically significant shift in the cline centre was detected towards the PEDC-PADC contact zone. This encompasses the main area of polymorphism, exhibiting an increased number of homozygous mice for the Rb (7.15) fusion over the last decade. Nevertheless, despite the change of the cline centre of Rb (7.15), the absence of a major geographical shift of this zone seems to exclude a possible numerical imbalance between the races, possibly related with a lower density of mice at the hybrid zone [17], allowing each population to evolve independently and 7.15 to increase towards fixation. A similar situation has been already described by Castiglia & Capanna [14] in a contact zone between two chromosomal races in Italy. Considering the observed narrowing of the contact zone after about 10 years, it would be interesting to address the dynamics of both chromosomal races and their hybrid zone after a longer timeframe.

### 4.2. Dynamics of the Contact Zone Between Estreito da Calheta (PEDC) and Achadas da Cruz (PADC) Races

Contact or hybrid zones are narrow regions where two more or less homogeneous parental forms meet and interbreed [39,40]. These zones are maintained by the influence of diverse factors, e.g., a balance between incompatibility of chromosomal races and dispersal [3,8,10,41,42,43,44]. This definition clearly fits the hybrid zone between PEDC and PADC chromosomal races. Two main mechanisms may determine patterns of gene flow between the hybridising taxa: i) ‘suppressed recombination’, contributing to the accumulation of genetic incompatibilities between parental types, and ii) ’hybrid dysfunction’, suggesting that hybrids or heterozygotes are less fit, and thus are selected against [8,44,45]. The role of both mechanisms has been discussed for different geographic metacentric ‘systems’ of the house mouse [8,44,46,47]. 

A single study on the fertility of hybrids between PEDC and PADC discussed the more traditional concept model of ‘hybrid dysfunction’. Results revealed that hybrids between these two races obtained under experimental conditions exhibited moderate subfertility (approximately a 50% decrease), suggesting that, under natural conditions, this level of underdominance could contribute to limit gene flow between parental populations [48]. 

A tension zone may move if there is a difference in population size (affecting dispersal rate) for the two races that are hybridising [42]. Nunes et al. [17], on the basis of the results of the 1998–2002 survey and on the distribution and abundance of crops and agricultural areas over the studied transect, suggested that potential habitats for mice are of better quality, more abundant and more evenly distributed over the area occupied by PEDC, while potential habitats for PADC are of poorer quality, fewer and more dispersed. This environmental contrast defines a source-sink continuum and could result in an asymmetrical dispersal rate into the contact zone between the two races [49,50]. In a source-sink system the contribution of sinks is indeed relevant for the persistence of individuals but a stable equilibrium between populations can be maintained in invariant environments [26]. According to this hypothesis, PEDC mice are expected to move more into/across the hybrid zone when compared with PADC mice. Despite a slight (non-significant) shift of the Rb (6.7) cline in that direction, further contributing to the observed narrowing of the contact zone, it was the Rb (7.15) fusion that significantly increased in frequency in the contact zone. Also, this increase may also be favoured by sexual selection as predicted in Nunes et al. [20], where patterns of preference of both males and females in the contact zone would favour Rb (7.15) individuals and thus contribute to the observed cline shift. Genomic analysis would be valuable in assessing the level and direction of gene flow across the hybrid zone, helping clarify the putative contribution of environmental quality on the dynamics of mouse movement across this area. 

The relative stability of the zone may also be explained by the fact that environmental conditions in this area did not change significantly. In fact, during the period of approximately 10 years, between the past and present survey, no marked changes were noticed. The types of crops and land use were almost the same in 2012-2014 as before, with no notable reduction in agricultural areas associated with urbanisation, suggesting environmentally stable conditions. Hence, we did not expect demographical changes both within and at the border of the hybrid zone. 

The role of commensalism of mice in combination with a fragmented landscape matrix and the extreme topography of Madeira has restricted the movement of mice and contributed to population isolation and fixation of fusions, namely the fixation of Rb (7.15) in the northernmost part of the transect [17,23]. 

The maintenance of the contact zone, although narrower, can also be explained by the persistence of an ‘acrocentric peak’, where mice carry acrocentric chromosomes 6, 7, and 15, limiting the production of hybrids (only two PEDC-PADC hybrids were identified in 1998–2002 and none in 2012-2014). There is an expectation that individuals that are acrocentric for chromosomes 6, 7, and 15 will be favoured because they cannot produce PEDC x PADC F_1_ hybrids and therefore will have more grand-offspring than other karyotypic categories. This situation is described by Nunes et al. [46 and references therein] and Gündüz et al. [5]. These authors suggest that the comparatively lower fitness of PEDC x PADC F_1_ hybrids, characterised by a chain-of-four configuration at meiosis I (6-6.7-7.15-15), will favour individuals homozygous for chromosomes 6, 7, and 15 thus leading to a high frequency of acrocentrics in the contact zone. On the other hand, meiotic drive may help to explain the slight but significant narrowing of the hybrid zone by maintaining metacentrics involving the fusions Rb (6.7) and Rb (7.15) in the area [51]. Natural variation in centromere strength has been found in wild populations of the house mouse, including in Madeira, where relatively stronger centromeres of metacentric chromosomes are preferentially retained in the egg in opposition to centromeres of acrocentrics, more likely to be segregated to the polar body [51]. As such, in these populations, where metacentrics tend to naturally accumulate, hybrid zones characterised by an ‘acrocentric peak’ may persist but become reduced in width, as observed in our study, reflecting the balancing forces in action as discussed above.

Thus, it seems reasonable to propose that multiple selective pressures (e.g., hybrid underdominance coincident with a peak of acrocentrics, meiotic drive, differences in habitat quality, habitat fragmentation) may be acting together resulting in the observed changes of the position and structure of the PEDC-PADC contact zone. 

### 4.3. Contact Zone Between Races Estreito da Calheta (PEDC) and Lugar de Baixo (PLDB)

In the present study, we also analysed to what extent landscape discontinuities constrain mouse migration and the karyotypic structure of the contact zone between PEDC and PLDB. These two races have very limited distributions, with the PLDB occupying a restricted area east of PEDC which in turn is parapatric with PADC as discussed above.

The chromosomal analysis of mice across the distributional areas of the two races shows a contact zone between them encompassing approximately 3 km, i.e., from km 7.2 to km 10.1 along the transect, with all hybrid individuals detected between both races located between km 8.48 and 10.1. 

It is worth pointing out that similarly to metacentrics Rb (6.7) and Rb (7.15), there was a possible role of WARTs in generating the diagnostic metacentrics Rb (5.18) and Rb (15.18) [4]. As such, it was not expected that chromosome 18, in particular, could occur here in an acrocentric form unless by migration of acrocentric-bearing mice, as inferred for the PEDC-PADC contact zone [5] or for contact zones between chromosomal races in Italy [7,14] and Spain [52].

The offspring resulting from the reproduction between mice of the PEDC and the PLDB races will most likely exhibit hybrid unfitness. Because races have metacentrics with monobrachial homology, the F_1_ between Rb (5.18) and Rb (15.18) form a chain-of-four at meiosis I, therefore, as for PEDC x PADC offspring, underdominance in hybrids can be anticipated [46]. Nevertheless, a surprisingly large number of hybrids were detected between both races across a much wider area when compared to the PEDC-PADC contact zone. Assuming that the 2km-wide band of non-commensal habitat is not an unbridgeable obstacle for active dispersal between the two races, in particular for PEDC mice, in addition to the possibility of passive dispersal movements, one can ask what mechanisms could have hampered the movement of PLDB mice across the valley. It would be interesting to further understand whether there is a sex-biased dispersion (expectedly by young males, [53]) into the PLDB race acting as a vehicle of gene flow contributing to the width of the contact zone between both races. Although the area inhabited by the PLDB race is occupied by sparse commensal habitat mostly surrounded by forests and woodlands, potentially offering fewer opportunities for mice to find shelter and food [54], whether this area can function as a sink as opposed to the PEDC source-area should be better investigated. In support of this hypothesis, a previous study showed that the amount of energy spent for maintenance is different between races and slightly lower in PLDB (energy intake: PLDB 28.96 ± 7.59 KJ day^−1^; PEDC 32.80 ± 2.64 KJ day^−1^) [55]. These differences in energy balance may reflect typically lower food availability for PLDB mice as a proxy for lower habitat quality [55,56]. This is again consistent with previous findings by Nunes et al. [17] regarding the PADC and PEDC races. Future studies will allow us to focus on a better understanding of the formation, maintenance, and evolution of this contact zone.

Most of the previously described hybrid zones in house mice involve a metacentric race and the all-acrocentric standard race [3]. The present data on the house mouse metacentric ‘system’ in Madeira reinforce the knowledge on the contact zones between different metacentric races. Further behavioural experiments coupled with genome-wide analysis will allow a better understanding of the underlying mechanisms maintaining the equilibrium of contact zones between Rb races. As such, it will be possible to assess levels of gene flow, its preferential direction (if any), identification of loci under selection, loci presenting clines across the contact zone, and its potential role in survival and reproduction [57,58].

## Figures and Tables

**Table 1:** Maximum likelihood estimates of cline centre position and cline width for Rb 6.7 (logistic regression model) and Rb 7.15 (scaled logistic regression model) and respective confidence intervals.

Clines	Centre	Confidence Interval	Width	Confidence Interval
**Rb 6.7 1998–2002**	19.07	18.37 - 19.76	12.48	10.70 - 14.95
**Rb 6.7 2012–2014**	18.26	17.22 - 19.30	10.61	8.73 - 13.52
**Rb 7.15 1998–2002**	24.71	24.16 - 25.26	2.09	1.49 - 3.52
**Rb 7.15 2012–2014**	23.89	23.45 - 24.33	1.96	1.19 - 5.52

## Supplementary Materials

The following are available online at https://www.mdpi.com/2073-4425/11/7/748/s1, Table S1: Description of localities sampled between 2012 and 2014 along a transect, including location name, identification code (ID), site number, GPS coordinates (Latitude and Longitude), distance along the transect (km) and number of house mice karyotyped (N), Table S2: Description of sites sampled between 1998 and 2002 including location name, identification code (ID), site number, GPS coordinates (Latitude and Longitude), distance along the transect (km) and number of animals karyotyped (N), Table S3: Maximum likelihood estimates and Akaike values computed on CFit8 considering eight cline models.
