# Supplementary material for: Spatial and Temporal Dynamics of Contact Zones Between Chromosomal Races of House Mice, Mus musculus domesticus, on Madeira Island
Source: Genes (Basel). 2020 Jul 6;11(7):748. doi: 10.3390/genes11070748 (PMC7397221; doi:10.3390/genes11070748)
Supplement: Supplementary file 1 [file genes-11-00748-s001.pdf]

genes

MDPI

Supplementary Material

Table S1. Description of localities sampled between 2012 and 2014 along a transect, including location name, identification code (ID), site number, GPS coordinates (Latitude and Longitude), distance along the transect (km) and number of house mice karyotyped (N).

Location	ID	Site	Latitude	Longitude	Distance	N
Ponta do Sol	P10	1	32.702385	−17.094955	2.06	4
Canhas	P86	2	32.705847	−17.113467	6.03	2
Salões	P88	3	32.700984	−17.121280	7.16	10
Socorro	P75	4	32.711636	−17.120763	8.40	5
Socorro	P76	5	32.711999	−17.122144	8.48	5
Sítio da Fajã	P36	6	32.713382	−17.131736	10.10	16
Moledos	P38	7	32.707210	−17.137733	12.00	2
Madalena - Arco da Cova da Calheta	P15	8	32.708986	−17.140775	12.52	3
Madalena do Mar	P11	9	32.711164	−17.140002	13.11	5
Arco da Calheta	P63	10	32.716946	−17.140532	13.47	7
Arco da Calheta (Cova do Calhau)	P58	11	32.722698	−17.139047	13.49	2
Arco da Calheta	P56	12	32.719466	−17.141714	13.73	8
Arco da Calheta	P57	13	32.719692	−17.142467	13.88	3
Arco da Calheta	P59	14	32.722987	−17.147405	14.36	2
Arco da Calheta	P60	15	32.723610	−17.146890	14.36	10
Arco da Calheta	P61	16	32.724697	−17.156718	16.04	10
Loreto	P01	17	32.723075	−17.153715	16.16	3
Arco da Calheta	P62	18	32.724645	−17.147777	16.63	3
Lombo do Doutor	P07	19	32.729530	−17.161975	19.85	2
Lombo do Doutor	P37	20	32.726480	−17.166846	20.53	3
Lombo do Salão	P35	21	32.737095	−17.166925	22.29	1
Lombo das Laranjeiras	P03	22	32.732179	−17.173866	23.08	5
Estreito da Calheta	P74	23	32.732130	−17.182553	25.36	14
Estreito da Calheta	P73	24	32.737529	−17.188818	27.27	6
Estreito da Calheta	P05	25	32.746573	−17.186641	28.60	2
Prazeres	P09	26	32.753293	−17.202090	31.37	1
Prazeres	P79	27	32.746874	−17.209961	31.39	7
Prazeres	P06	28	32.754545	−17.201605	31.39	1
Solar dos Prazeres	P77	29	32.752394	−17.209188	31.89	7
Lombo da Velha	P44	30	32.758306	−17.204102	31.98	5
Maloeira - Rua do Pico	P78	31	32.759179	−17.212979	32.95	4
Maloeira	P13	32	32.762066	−17.211698	32.95	2
Maloeira	P45	33	32.766396	−17.208624	33.00	4
Maloeira	P12	34	32.768473	−17.206542	33.42	15
Solar da Maloeira	P46	35	32.770350	−17.205491	33.47	2
Lugar da Raposeira	P81	36	32.762332	−17.217083	34.17	17
Lugar da Raposeira	P80	37	32.762549	−17.220505	34.18	3
Lombada dos Cedros	P14	38	32.774626	−17.215009	35.24	1
Fajã da Ovelha	P43	39	32.776904	−17.238386	38.88	3
Lombada dos Marinheiros	P19	40	32.788776	−17.234668	40.50	9
Ponta do Pargo	P67	41	32.808734	−17.241250	45.22	9
Ponta do Pargo	P66	42	32.812078	−17.251840	46.09	11
Ponta do Pargo	P41	43	32.812677	−17.251086	46.09	5
Ponta do Pargo	P64	44	32.813313	−17.249471	46.11	6
Ponta do Pargo	P40	45	32.813856	−17.249812	46.13	6
Ponta do Pargo - Ribeira da Vaca	P20	46	32.819506	−17.243423	47.56	2
Ribeira da Vaca	P65	47	32.817656	−17.237900	48.31	18
Maloeira	P17	48	32.818857	−17.227589	49.54	3
Achadas da Cruz	P39	49	32.843201	−17.210391	57.83	4

Achadas da Cruz	P30	50	32.840862	−17.207079	58.17	10
Santa	P31	51	32.857735	−17.181673	64.27	4
Levada Grande	P71	52	32.856734	−17.169571	66.28	2
Porto Moniz	P32	53	32.864023	−17.174399	68.39	2
Ribeira da Janela	P33	54	32.848303	−17.153558	72.64	5
Ribeira da Janela	P72	55	32.844618	−17.156318	72.72	4
Ribeira Funda	P25	56	32.829188	−17.134002	75.59	6
Ribeira da Laje	P24	57	32.826122	−17.118856	77.36	4
Seixal - Sitio da Portada	P23	58	32.822270	−17.106770	79.12	1
Chão da Ribeira	P22	59	32.819802	−17.109307	79.83	4
Chão da Ribeira	P21	60	32.811940	−17.111600	81.61	6
Chão da Ribeira	P70	61	32.810472	−17.113068	81.81	13
Chão da Ribeira	P68	62	32.811313	−17.113733	81.81	12
Chão da Ribeira	P83	63	32.809056	−17.114917	82.16	3
Chão da Ribeira	P82	64	32.806284	−17.113317	82.50	1
Chão da Ribeira	P69	65	32.804288	−17.113110	82.78	8

Table S2. Description of sites sampled between 1998 and 2002 including location name, identification code (ID), site number, GPS coordinates (Latitude and Longitude), distance along the transect (km) and number of animals karyotyped (N).

Location	ID	Site	Latitude	Longitude	Distance	N
Lugar de Baixo	64	1	32.679893	−17.091101	0	8
Santo Amaro	106	2	32.680725	−17.099880	1.300	6
São Caetano	107	3	32.684811	−17.100710	2.400	6
Canhas	114	4	32.694424	−17.110970	5.910	16
Salões	113	5	32.701611	−17.124410	7.580	5
Fajã	116	6	32.713438	−17.131930	10.103	1
Moledos	108	7	32.709694	−17.136660	11.680	17
Palheiros	131	8	32.714585	−17.141100	13.373	6
Amoreiras	109	9	32.718009	−17.144040	13.870	20
Lombada do Loreto	110	10	32.725928	−17.153110	16.893	16
Lombo da Atouguia	111	11	32.728666	−17.159090	19.556	9
Lombo do Salão	112	12	32.731597	−17.170970	22.984	13
Calheta	119	13	32.721052	−17.177320	23.669	3
Estreito da Calheta	69	14	32.739108	−17.183020	26.582	4
Sítio dos Reis	68	15	32.739179	−17.183220	26.598	14
Sítio dos Moinhos Abaixo	115	16	32.735329	−17.191470	27.268	22
Venda do Atalhinho/Cima	118	17	32.744265	−17.191830	28.194	12
Jardim do Mar	117	18	32.737527	−17.211720	31.373	4
Lombo da Carreira	122	19	32.751771	−17.210310	31.887	22
Prazeres	71	20	32.758757	−17.203310	31.952	19
Prazeres	70	21	32.758902	−17.203480	31.980	12
Lombo da Rocha	121	22	32.756289	−17.209380	32.206	28
Lombo do Coelho	120	23	32.766200	−17.202210	32.629	31
Maloeira	123	24	32.767452	−17.205900	32.775	20
Lombo da Velha	72	25	32.775915	−17.201740	33.465	11
Paúl do Mar	74	26	32.757790	−17.229229	34.174	7
Paúl do Mar	73	27	32.757210	−17.229526	34.184	3
Lombada dos Cedros	130	28	32.776782	−17.212960	35.136	19
Lombada dos Cedros	126	29	32.773272	−17.217190	35.401	2
Sítio da Lombada dos Cedros	127	30	32.773717	−17.218520	35.429	16
Lombada dos Marinheiros	125	31	32.790022	−17.235690	40.624	18
Sitio do Lombo	76	32	32.793336	−17.235230	41.508	4
Sítio do Lombo	129	33	32.796518	−17.234590	42.018	5
Lombo	124	34	32.796358	−17.237270	42.178	17
Serrado	135	35	32.818511	−17.241170	47.945	18
Serrado	137	36	32.818863	−17.239810	48.207	4

Ribeira da Vaca	134	37	32.820648	−17.231010	49.656	15
Ribeira da Vaca	77	38	32.820461	−17.231300	49.682	1
Achadas da Cruz	79	39	32.841130	−17.211973	57.443	1
Santa	45	40	32.860314	−17.191034	63.201	2
Santa	44	41	32.859610	−17.190746	63.225	1
Fajã da Parreira	48	42	32.826248	−17.120079	77.224	1
Seixal	56	43	32.822943	−17.105693	79.116	1
Chão da Ribeira	39	44	32.820104	−17.108419	79.831	2
Chão da Ribeira	136	45	32.820089	−17.108405	79.831	19
Chão da Ribeira	49	46	32.811720	−17.111589	81.634	1
Chão da Ribeira	139	47	32.811769	−17.113412	81.787	11

Table S3. Maximum likelihood estimates and Akaike values computed on CFit8 considering eight cline models.

		**Rb 6.7 1998-2002**		**Rb 6.7 2012-2014**	
**Models**	**Parameters**	**llmax**	**AIC**	**llmax**	**AIC**
Logit	2	−249.838991	503.678	−80.30860387	164.617
ScaledLogit	3	−249.838991	505.678	−78.76242812	163.525
RALogit	3	−265.4618194	536.924	−108.7637537	223.528
ScaledExp	3	−300.8838798	607.768	−122.652364	251.305
ScaledSquaredExp	3	−14247.58405	28501.2	-11824.33881	23654.7
H1H2ScaledLogit	4	−249.3928437	506.786	−78.76242812	165.525
ScaledMixedExp	4	−14247.57932	28503.2	−11824.36967	23656.7
ScaledRALogit	4	−254.7550029	517.51	−91.75784575	191.516

		**Rb 7.15 1998-2002**		**Rb 7.15 2012-2014**	
**Models**	**Parameters**	**llmax**	**AIC**	**llmax**	**AIC**
ScaledLogit	3	−126.4874322	258.975	−88.27436304	182.549
Logit	2	−152.2481569	308.496	−89.76127952	183.523
RALogit	3	−139.8702205	285.74	−98.76707459	203.534
ScaledExp	3	−153.1556179	312.311	−90.78348754	187.567
ScaledSquaredExp	3	−6606.351319	13218.7	−1677.540892	3361.08
H1H2ScaledLogit	4	−126.4874322	260.975	−88.27436304	184.549
ScaledMixedExp	4	−6606.436047	13220.9	−1677.540914	3363.08
ScaledRALogit	4	−127.2757685	262.552	−88.46605009	184.932
